# Impact of Elevated Screen Time on School-Age Adolescents During the COVID-19 Pandemic: An Analytical Study

**DOI:** 10.7759/cureus.64689

**Published:** 2024-07-16

**Authors:** Roshni Manuja, Tanuja P Pattankar, Mallikarjun C Yadavannavar, Rekha S Udgiri

**Affiliations:** 1 Community Medicine, Shri Basangouda Mallangouda (BM) Patil Medical College, Bijapur Lingayat District Educational (BLDE) Deemed to be University, Vijayapura, IND

**Keywords:** covid-19, health, pandemic, adolescents, screen time

## Abstract

Introduction: The rise in digital technology use, especially during the COVID-19 pandemic, has heightened concerns about extended "screen time" (ST) among adolescents. Excessive screen use is linked to negative physical and cognitive effects, yet digital media also offers educational and social advantages. Cultivating healthy digital habits is crucial as technology continues to evolve.

Methodology: This cross-sectional study was conducted between July and August 2022 among school-going adolescents and a total of 221 participants were assessed through questionnaires, employing statistical analysis.

Results: In the current study, all participants fell within the 16-18 year age group, with 114 (51.6%) being male and 107 (48.4%) female. The majority 209 (94.5%) identified as Hindu, while the rest were from Muslim and Christian communities. Most participants, both male and female, resided in urban areas, 140 (63.3%) were pursuing Class 12, and 73 (33.1%) were pursuing Class 11. Mobile phones were the most commonly owned devices, followed by desktops or laptops and tablets. Males generally spend more time on these devices than females. Notably, 168 (76.01%) adolescents exceeded the recommended 2-hour daily screen time limit set by the American Academy of Pediatrics (AAP), with 92 (80.7%) males more likely to do so than 76 (71%) females. The study found a significant link between increased screen time, reduced physical activity, and academic performance, while no association was observed for behavioral risk factors.

Conclusion: There is increased digital device use among youth, with many exceeding recommended screen time limits, particularly males. This was significantly linked to reduced physical activity and academic decline, highlighting the need for further research and balanced digital use.

## Introduction

In recent years, there has been a notable rise in the use of digital technology, resulting in greater accessibility to various screen-based media devices such as smartphones, laptops, and tablets among adolescents. This has led to a significant increase in the average duration of screen time (ST) exposure in this demographic, raising serious concerns [1]. The COVID-19 pandemic and subsequent measures like stay-at-home orders, online learning, and social distancing, implemented between 2020 and 2022, have further heightened adolescents' dependence on digital media devices including mobile phones, tablets, iPads, laptops, and televisions [2]. Adolescents now rely on these devices for entertainment, social interaction, and education [3].

The concept of screen time (ST) has thus become more complex, encompassing a broad range of screen-based devices. Studies have demonstrated that excessive ST is negatively related to physical and cognitive development and is positively linked to health issues such as obesity, sleep disturbances, depression, and anxiety [4]. On the other hand, the use of digital and social media among adolescents has evidence-based benefits, including early learning opportunities, exposure to new ideas and knowledge, enhanced social interactions, and better access to health information [5]. With the continuous growth in the demand for technology, the consequences of prolonged ST remain mixed, making it increasingly difficult to distinguish between healthy and unhealthy social connectivity through digital media. Therefore, promoting healthy digital habits is essential, especially since digital technology is expected to remain a key part of our lives and continue to evolve [6].

This study aimed to address the scarcity of research on the effects of increased ST on the physical, behavioral, and academic performance of school-going adolescents during the ongoing COVID-19 pandemic.

## Materials and methods

This cross-sectional study was conducted in July and August 2022 in North Karnataka, India. The study population comprised school-going adolescents aged 16-18 years. The sample size was determined by using the anticipated mean±SD of ST of adolescents, which was 7.70±5.74 h/day according to a previous study [7], hence the study would require a sample size of 202 adolescents with a 95% level of confidence and a precision of 0.8, the formula used was n=z^2 ^× s^2^/d^2^ [8]. Where Z is Z statistic at α level of significance, d is absolute error, and S is common standard deviation. The total sample collected was 221.

Following institutional ethical clearance from BLDE Deemed to be University under the reference number #BLDE (DU)/IEC/631-B/2022-23, the sample size was determined using a probability sampling technique. For this study, two schools were selected via simple random sampling, consisting of one private school and one private aided school. The sample included 111 students from private schools and 110 students from private-aided schools, both chosen through systematic random sampling. With the necessary permissions and consent secured from the principals or headmasters of the respective schools, the study was initiated. Students who agreed to participate were included, while those absent for three consecutive visits or unwilling to participate were excluded.

Data was collected using validated tools, specifically the Global Physical Activity Questionnaire (GPAQ) for physical activity measurement [9], the Questionnaire for Screen Time of Adolescents (QueST) for assessing screen time in adolescents [10], and for assessing additional sociodemographic data a semi-structured self-administered questionnaire was utilized. These questionnaires are provided in the appendices, appendix 1 (GPAQ) and appendix 1 (QueST). This systematic methodology enabled an in-depth analysis of screen time and its related factors among school-going adolescents in North Karnataka.

For statistical analysis, the data obtained was entered in a Microsoft Excel sheet (Redmond, WA: Microsoft Corp.), and statistical analysis was performed using SPSS Statistics for Windows, version 20.0, released in 2011 (Armonk, NY: IBM Corp.). Results are presented as mean (median)±SD, counts, percentages, and diagrams. Categorical variables are compared using the chi-square test and Student's t-test was used for continuous variables. P<0.05 is considered statistically significant.

## Results

In this study, we included adolescents aged 16, 17, and 18 years, with seven (3.1%), 106 (48.0%), and 108 (49%), respectively. The gender distribution comprised 114 (51.6%) males and 107 (48.4%) females. Of the participants, 209 (94.5%) identified as Hindu and 204 (92.3%) resided in urban areas. The majority were pursuing Class 12 (140 or 63.3%) and Class 11 courses (73 or 33.1%) (Table 1).

**Table 1:** Sociodemographic profile of study participants.

Variables		n (%)
Age	16 years	7 (3%)
	17 years	106 (48%)
	18 years	108 (49%)
Gender	Male	114 (51.6%)
	Female	107 (48.4%)
Religion	Hindu	209 (94.5%)
	Muslim	8 (3.6%)
	Christian	4 (1.8%)
Place of living	Urban	204 (92.3%)
	Rural	17 (7.7%)
Education	Class 10	8 (3.6%)
	Class 11	73 (33.0%)
	Class 12	140 (63.3%)

**Figure 1:**
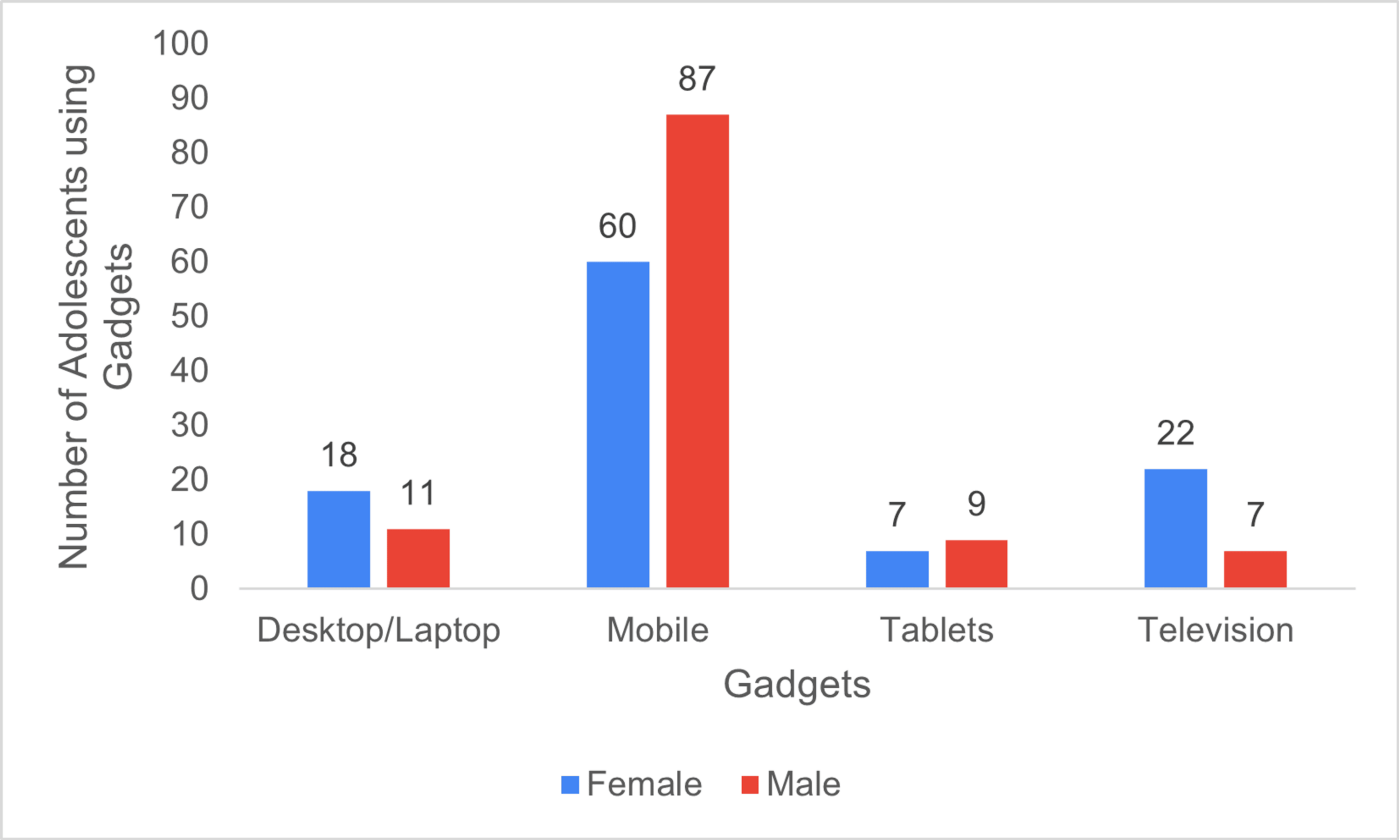
Distribution of gadgets owned by study participants.

On assessing usage of screen-based devices, we found most adolescents owned mobile phones, with 87 (76.3%) males and 60 (56.1%) females owning one. Ownership of desktops or laptops was reported by 18 (16.8%) females and 11 (9.6%) males, while seven (6.5%) females and nine (7.9%) males had tablets. A smaller number of participants used only television for entertainment as follows: 22 (20.5%) females and seven (6.1%) males (Figure 1).

The average screen time was higher for males (4.35±2.21 h/day) compared to females (3.88±1.85 h/day). No significant statistical differences were found between genders, except for entertainment, gaming, and social media usage (Table 2).

**Table 2:** Distribution of means and SDs to assess the difference in usage of screen-based devices among different genders. *P-value<0.05 was considered statistically significant.

Variables	Female (n=107)			Male (n=114)			t-test p-value
	Mean	Standard deviation	Standard error of mean	Mean	Standard deviation	Standard error of mean	
Total time spent on screen-based devices (each day)	3.88	1.85	0.18	4.35	2.21	0.21	0.088
Usage of screen-based devices for studies	1.9	2.1	0.2	1.89	2.1	0.19	0.98
Usage of screen-based device for internship/work	0.82	1.82	0.17	0.76	1.23	0.11	0.726
Usage of screen-based devices for entertainment	2.03	1.4	0.13	1.62	1.05	0.09	0.016^*^
Usage of screen-based devices for gaming and social media	1.9	1.67	0.16	2.98	1.68	0.15	0.0001^*^

When we assessed the effect of increased screen time on physical activity and academic performance, we found a significant majority, 182 (82.4%), believed their screen time had increased during the pandemic, and most reported they could not abstain from screen devices for a day. Those who could abstain chose to engage in activities like outdoor games, music, dancing, homework, drawing, painting, or spending time with family (Table 3).

**Table 3:** Distribution of study participants on their perception regarding usage of screen-based devices.

Variables	Responses	Frequency	%
Do you think your usage of screen-based devices has increased post-COVID-19?	Yes	182	82.4
	No	39	17.6
Can you stay without using these gadgets for a day?	Yes	83	37.6
If yes, then what other activities do you involve yourself?	Games	19	22.9
	Music	19	22.9
	Dance	10	12.0
	Drawing and painting	5	6.0
	Family time	4	4.8
	Homework	12	14.5
Can you stay without using these gadgets for a day?	No	138	62.4
Why do you think you cannot stay without using them?	Boredom	106	76.8
	Feeling of low	15	10.9
	Is your studies/homework affected without using them?	36	26.1
	Not interested in any other activities	49	35.5

**Figure 2:**
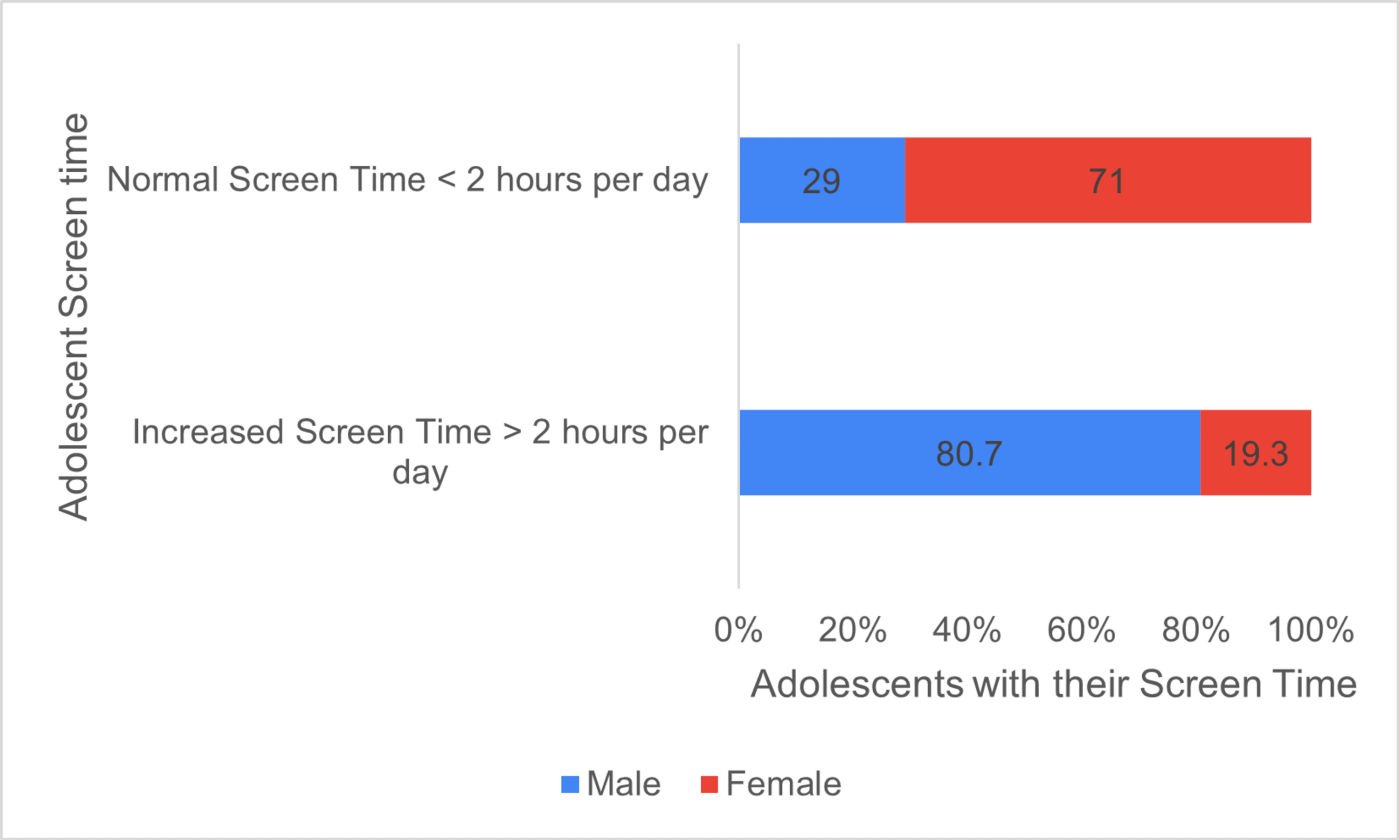
Percentage of adolescents having screen time >2 h per day (increased) and <2 h per day (normal).

It was alarming to note that among all the study participants 92 (80.7%) males and 31 (19.3%) females exceeded the American Academy of Pediatrics' recommendation for screen time of 2 h per day (Figure 2). We found that increased screen time significantly negatively affected physical activity and time spent with family and friends (Tables 4-6).

**Table 4:** Assessment of effect of increased screen time on physical activity. *P-value<0.05 was considered statistically significant. Increased screen time (>2 h/day) and normal screen time (<2 h/day).

Type of PA	Increased screen time (n=168)		Normal screen time (n=53)		Chi-square test
	n	%	n	%	<0.001^*^
Heavy vigorous activity	14	8.3	19	35.8	
Moderately vigorous activity	80	47.6	30	56.6	
Sedentary time spent	74	44.1	4	7.5	

**Table 5:** Assessment of difference in mean academic scores of study participants before and after the COVID-19 pandemic. *P-value <0.05 was considered statistically significant.

Gender	Academic score before COVID-19 (out of 100)			Academic score after COVID-19 (out of 100)			t-test (p-value)
	Mean	Standard deviation	Standard error of mean	Mean	Standard deviation	Standard error of mean	
Male	54.7	11.4	1.068	50.8	12.1	1.133	0.013*
Female	68.4	10.6	1.025	61.6	11.8	1.41	0.0001*

**Table 6:** Perception of study participants towards academic performances, physical activity, and time spent with family and friends due to increased ST post-COVID-19 pandemic. *P-value <0.05 was considered statistically significant. ST: screen time

Variables	Male (114)		Female (107)		Chi-square	p-Value
	n	%	n	%		
Academic performance reduced post-COVID-19 pandemic	58	50.9	64	59.8	1.78	0.23
Physical activity reduced post-COVID-19 pandemic	61	53.5	43	40.2	3.93	0.05^*^
Time spent with family and friends reduced post-COVID-19 pandemic	23	20.2	35	32.7	4.48	0.03^*^

**Figure 3:**
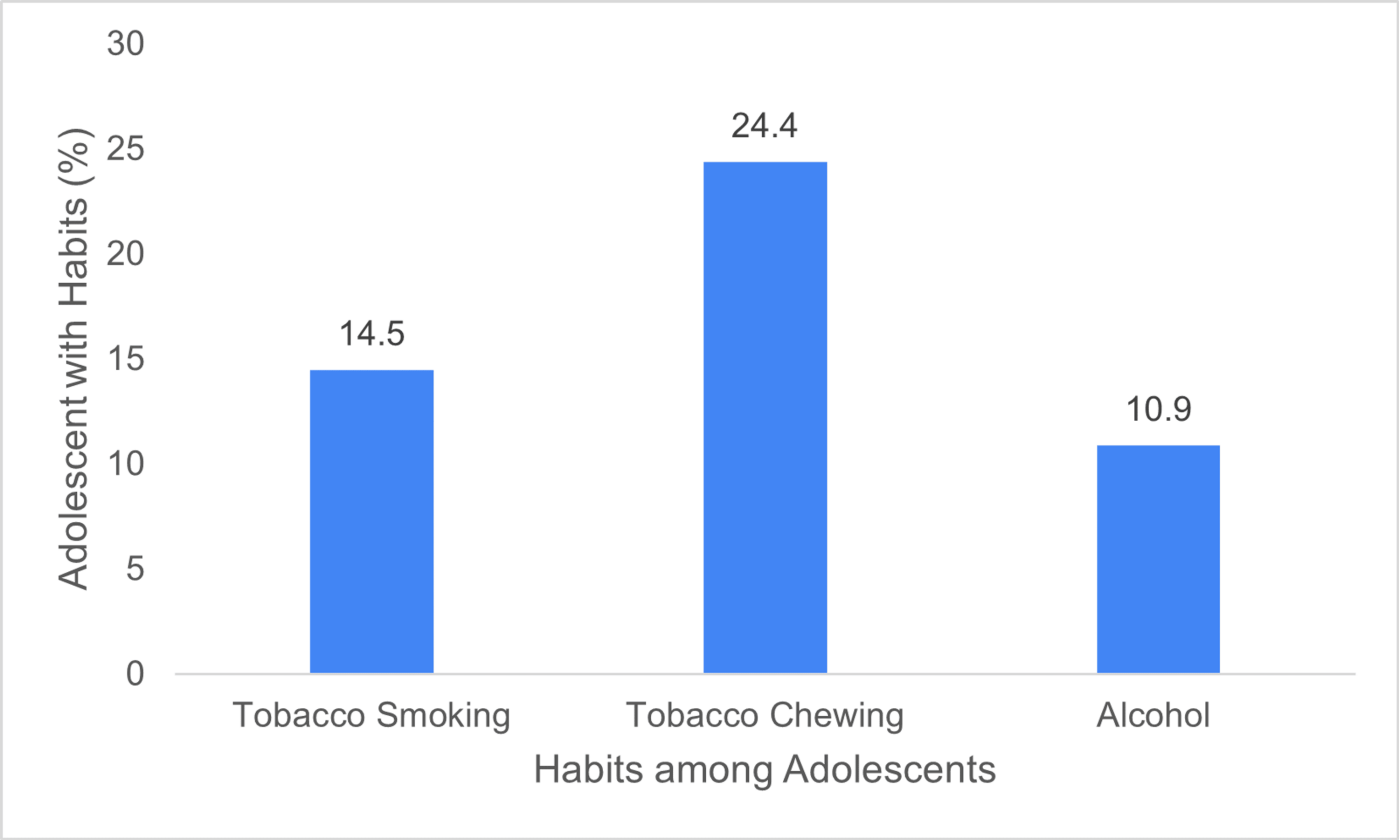
Percentage of the study population with habits.

The mean academic score for male students decreased from 54.7 before COVID-19 to 50.8 after COVID-19. The standard deviation slightly increased, indicating a wider dispersion of scores post-COVID-19. The p-value suggests the difference of means was statistically significant, showing that the pandemic had a significant negative impact on the academic performance of male students as shown in Table 5. Additionally, a small percentage of male adolescents reported engaging in tobacco and alcohol consumption (Figure 3).

**Figure 4:**
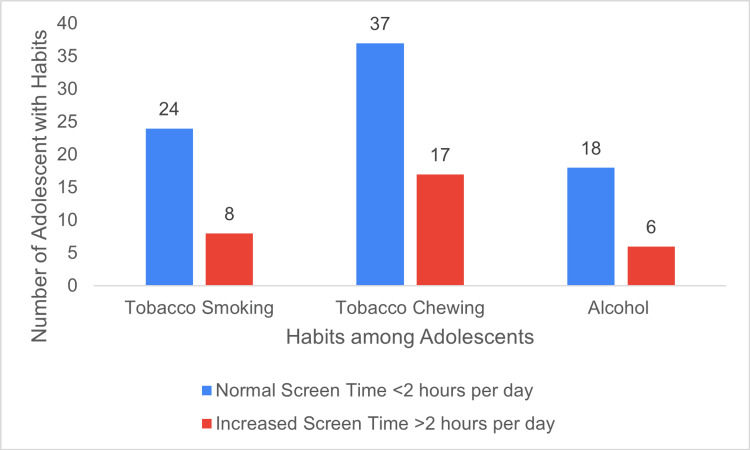
Assessment of behavioral habits among adolescents with increased ST and normal ST.

No significant differences were observed in behavioral habits between adolescents with increased screen time and those with normal screen time (Figure 4).

## Discussion

This research investigated the impact of increased screen time (ST) on school-going adolescents aged 16-18 years during the COVID-19 pandemic. Among the 221 participants, 147 (66.5%) owned mobile phones, 29 (13.2%) had desktops or laptops, and 16 (7.2%) had tablets, with an average ST of 4.1±2.01 h/day, which is notably higher than figures from previous studies in India. The participants were primarily students in Class 10 (SSLC), Class 11 (Pre-University I), and Class 12 (Pre-University II), as younger adolescents were excluded due to school regulations preventing those below Class 10 from using personal electronic devices.

A similar study by Dubey et al. in New Delhi found 98% of adolescents using screen-based media, consistent with our findings, but with a higher prevalence of television usage (96.5%), contrasting with our study where mobile phones were more common [11]. Dubey et al. reported an average screen time of 3.8 (±2.77) h/day, lower than our findings, likely because their data was collected before the COVID-19 pandemic in 2018.

According to the American Academy of Pediatrics (AAP), individuals older than two years should limit their screen time to no more than 2 h/day [12]. Our study found that 182 (82.4%) adolescents perceived an increase in their screen time during the pandemic, and 168 (76.01%) actually experienced this increase. These figures are significantly higher than those reported in a 2015 multicenter study by LeBlanc et al., which documented a 31% increase in India, 34% in China, 45% in Canada, and 59% in the United States [13].

Screen time was assessed using the QueST questionnaire, which is a five-item tool tailored for adolescents [10]. The mean screen time was 4.1±2.01 h/day, with males averaging 4.35±2.21 h and females 3.88±1.85 h. Males spent more time across various activities, including studies, internships/work, entertainment, gaming, and social media browsing compared to females.

Physical activity was measured using the GPAQ questionnaire [9]. Results showed that only 33 (14.9%) adolescents participated in heavy vigorous activity, averaging 3.07 days/week and 2.31 h/day. Additionally, 110 (49.8%) engaged in moderately vigorous activities, while 78 (35.5%) had a sedentary lifestyle. Compared to a study by Panchali Moitra and Madan, which found only 12% engaged in moderate to vigorous physical activity during the pandemic, our study showed a higher percentage [14]. This discrepancy may be due to varying COVID-19 restrictions at the time of each study.

Academic performance was evaluated by comparing percentage marks from final exams before and after the pandemic. A significant decline in mean percentage marks was observed, indicating reduced academic performance among both male and female participants. This decline was statistically significant, with 50.9% of males and 59.8% of females attributing their reduced academic performance to increased screen time. This finding aligns with a study by Paulich et al., which found that increased screen time was moderately associated with poorer mental health, behavioral problems, decreased academic performance, and poorer sleep, though it also noted improved quality of peer relationships [15].

Our study also found a statistically significant correlation between increased screen time and both poor physical activity and reduced academic performance. However, no significant association was found between increased screen time and behavioral risk factors among adolescents.

The COVID-19 pandemic has highlighted the need to reevaluate the concept of screen time from a health perspective, given the reliance on screen time as a measure for the healthy use of internet-enabled devices. Notably, the WHO has suggested online social networking to connect with peers and relatives during the pandemic [16]. This view is supported by the American Academy of Child and Adolescent Psychiatry’s statement on media habits and sedentary screen time during the COVID-19 quarantine among children and adolescents [17]. The AAP guidelines emphasize the importance of the content and context of digital media use by children [3]. A recent United Nations Children's Fund (UNICEF) report underscores that while the internet and digital technology play positive roles in education and leisure for children, there are concerns and potential risks associated with their use [18]. Another UNICEF report pointed out gaps and methodological limitations in evidence supporting arbitrary screen time cutoffs in the digital age [19]. It has been argued that the activities and content children and adolescents engage with through digital technology are likely more important than the amount of screen time in determining outcomes [20].

This study was limited to school-going adolescents, which may introduce bias, as the increase in screen time likely affects all adolescents, including those both attending school and those who have dropped out.

## Conclusions

The prevalence of digital media device use among children and adolescents has witnessed a significant and continuous rise. This study underscored a concerning trend, with a substantial proportion of adolescents exceeding the recommended daily screen time limit of 2 h, primarily utilizing mobile phones, laptops, and tablets. Notably, male participants exhibited greater screen time compared to their female counterparts, although this gender difference did not yield a statistically significant association. Of particular concern, our study revealed a statistically significant relationship between increased screen time, reduced physical activity, and a decline in academic performance. This study highlights the pressing need for further research, ideally of a longitudinal nature, to explore the long-term effects of extensive digital device use among adolescents. Examining these patterns across diverse socioeconomic strata would provide a more comprehensive understanding of the extent of media overuse.

## Appendices

Appendix 1

**Table 7:** GPAQ questionnaire for assessing general physical activity. GPAQ: Global Physical Activity Questionnaire

S. no.	Questions	Answers		
1	Do you walk or use a bicycle for at least 10 mins continuously to get to and from places?	Yes	No	If yes, then answer next two questions.
	In a typical week, how many days do you walk or bicycle for at least 10 mins continuously to get to and from places?	---------------- Days		
	How much time do you spend walking or traveling on a typical day?	------------------ Hours		
2	Do you do any vigorous-intensity sports, ﬁtness or recreational activities that cause large increases in breathing or heart rate like (running, football, or basketball) for at least 10 minutes continuously?	Yes	No	If yes, then answer next two questions.
	In a typical week, how many days do your vigorous-intensity sports, ﬁtness, or recreational activities?	---------------- Days		
	How much time do you spend on vigorous-intensity sports ﬁtness or recreational activities on a typical day?	------------------ Hours		
3	Do you do any moderate-intensity sports ﬁtness or recreational activities that cause a small increase in breathing or heart rate such as brisk walking (cycling, swimming, volley) for at least 10 minutes continuously?	Yes	No	If yes, then answer next two questions.
	In a typical week, on how many days do you do moderate-intensity sports, ﬁtness, or recreational activities?	---------------- Days		
	How much time do you spend doing moderate-intensity sports, ﬁtness, or recreational activities on a typical day?	------------------ Hours		
3	How much time do you usually spend sitting or reclining on a typical day?	------------------ Hours		

Appendix 2

**Table 8:** QueST questionnaire for assessing screen time. QueST: Questionnaire for Screen Time of Adolescents

On average how much time do you spend on using these devices?	On a weekday in hours	On a weekday in minutes
Studying: studying, watching video classes, reading, doing research, or schoolwork on a computer, television, tablet, smartphone, or other screen-based devices.		
Performing work/internship-related activities: doing job or internship-related work on a computer, television, tablet, smartphone, or other screen-based devices.		
Watching videos: watching TV shows, movies, soap operas, news, sports, programs, or other videos on a computer, television, tablet, smartphone, or other screen-based devices.		
Playing video games: playing video games on a games console, computer, television, tablet, smartphone, or other screen-based devices.		
Using social media/chat applications.		
